# Right paraduodenal hernia caused by a long vessel-like peritoneal band: a rare anatomical variation in a young adult

**DOI:** 10.1093/jscr/rjag388

**Published:** 2026-05-29

**Authors:** Abdulaziz AlKanhal, Osama Almubadel, Ammar Alzahim, Abdulrahman Alzamil, Maher Alsaadi, Mohannad Aladawi, Abdulrhman Aleisa

**Affiliations:** Trauma and Acute Care Surgery Unit, Department of Surgery, King Saud University, King Khalid Road, Diriyah District, Riyadh 11416, Riyadh Province, Saudi Arabia; College of Medicine, King Saud University, PO Box 2925 Riyadh 11461, Riyadh Province, Saudi Arabia; Department of Surgery, Prince Mohammed bin Abdulaziz Hospital, 9259 Al Wahah, Al Rawabi District, Riyadh 14214–3971, Riyadh Province, Saudi Arabia; Department of Surgery, Prince Mohammed bin Abdulaziz Hospital, 9259 Al Wahah, Al Rawabi District, Riyadh 14214–3971, Riyadh Province, Saudi Arabia; Department of Surgery, Prince Mohammed bin Abdulaziz Hospital, Al-Imam Ahmad Bin Hanbal Street, Ar Rawabi District, Riyadh 14214, Riyadh Province, Saudi Arabia; Trauma and Acute Care Surgery Unit, Department of Surgery, King Saud University, King Khalid Road, Diriyah District, Riyadh 11416, Riyadh Province, Saudi Arabia; Assistant Professor, Body Imaging, Abdominal and Pelvic Radiology Unit, Department of Radiology, College of Medicine, King Saud University, King Khalid Road, Diriyah District, Riyadh 11416, Riyadh Province, Saudi Arabia

**Keywords:** right paraduodenal hernia, internal hernia, small bowel obstruction, computed tomography, laparoscopic surgery

## Abstract

Paraduodenal hernias are rare congenital internal hernias, with right-sided cases being particularly uncommon and often difficult to diagnose due to nonspecific symptoms. We report a 21-year-old male with a 1-year history of recurrent abdominal pain and vomiting, previously managed conservatively. Physical examination and laboratory findings were unremarkable. Computed tomography demonstrated clustered small bowel loops in the right hemiabdomen with displacement of the superior mesenteric vessels, suggestive of an internal hernia. Laparoscopy confirmed a right paraduodenal hernia caused by a congenital mesenteric defect, which was successfully repaired laparoscopically, with prophylactic appendectomy performed. The patient had an uneventful recovery. This case highlights the importance of considering internal hernias in young patients with recurrent, unexplained abdominal symptoms and the role of early imaging and surgical intervention in preventing complications.

## Introduction

Internal hernia is defined as the protrusion of a viscus through a peritoneal or mesenteric aperture. Its incidence ranges between 0.5% and 0.9% and it is associated with high mortality rates, reaching over 50% by causing ~5.8% small bowel obstructions [1, 2]. Internal hernias can be classified based on the origin of the defect as either congenital or acquired, or based on the anatomical location of the defect [3]. Paraduodenal hernias are the most common type of congenital internal hernias, accounting for ~53% of all cases. They are further classified into left and right paraduodenal hernias, with the right type accounting for ~25% [4]. We present a rare case of a right paraduodenal hernia in a young adult male, diagnosed after a year of intermittent, nonspecific gastrointestinal symptoms. The hernia was confirmed by computed tomography (CT) imaging and successfully treated with laparoscopic surgery. This case underscores the diagnostic challenge posed by right paraduodenal hernias and the importance of considering internal hernias in patients with recurrent, unexplained abdominal symptoms.

## Case presentation

**Figure 1:**
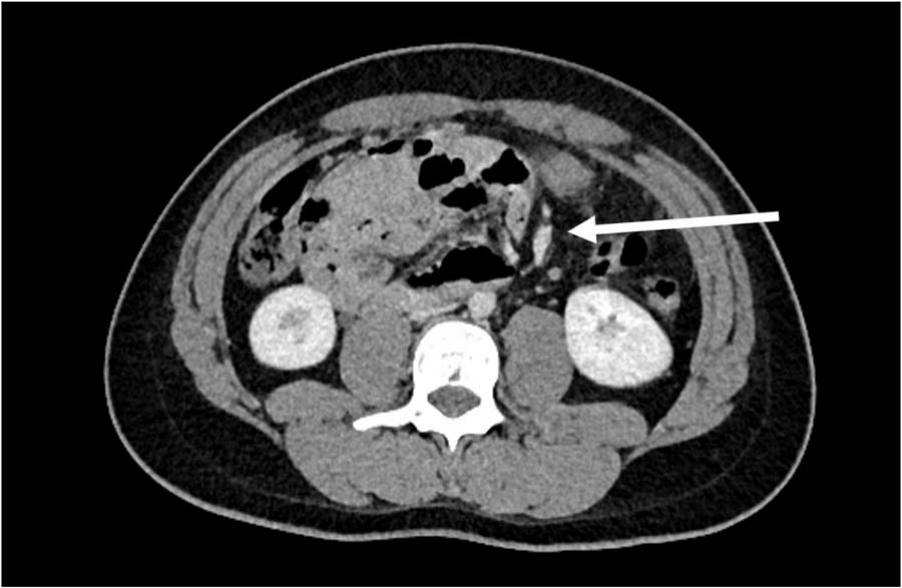
Axial CT image showing the clustered small bowel loops pushing the superior mesenteric vessels toward the left hemiabdomen (arrow points to the superior mesenteric vessels).

**Figure 2:**
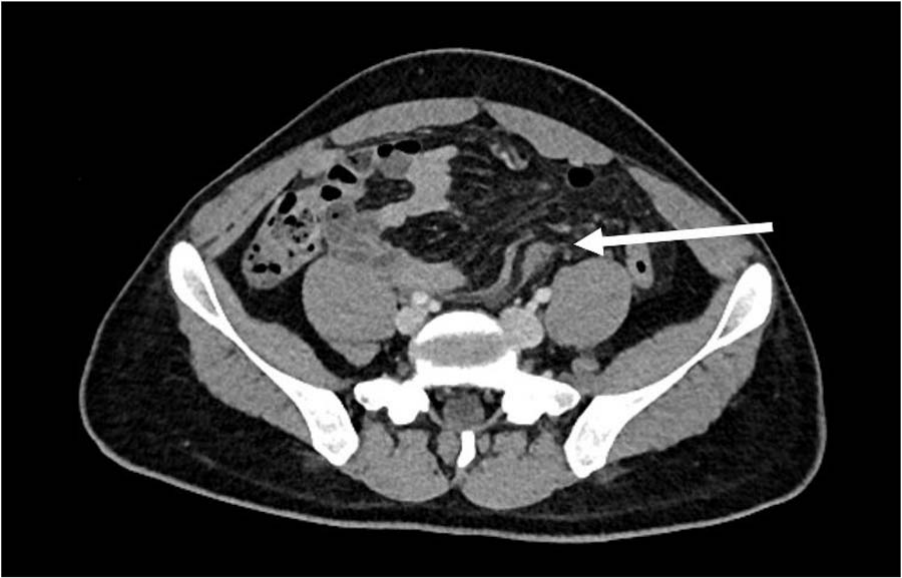
Axial CT image showing the thick band near the iliac vessels (arrow).

**Figure 3:**
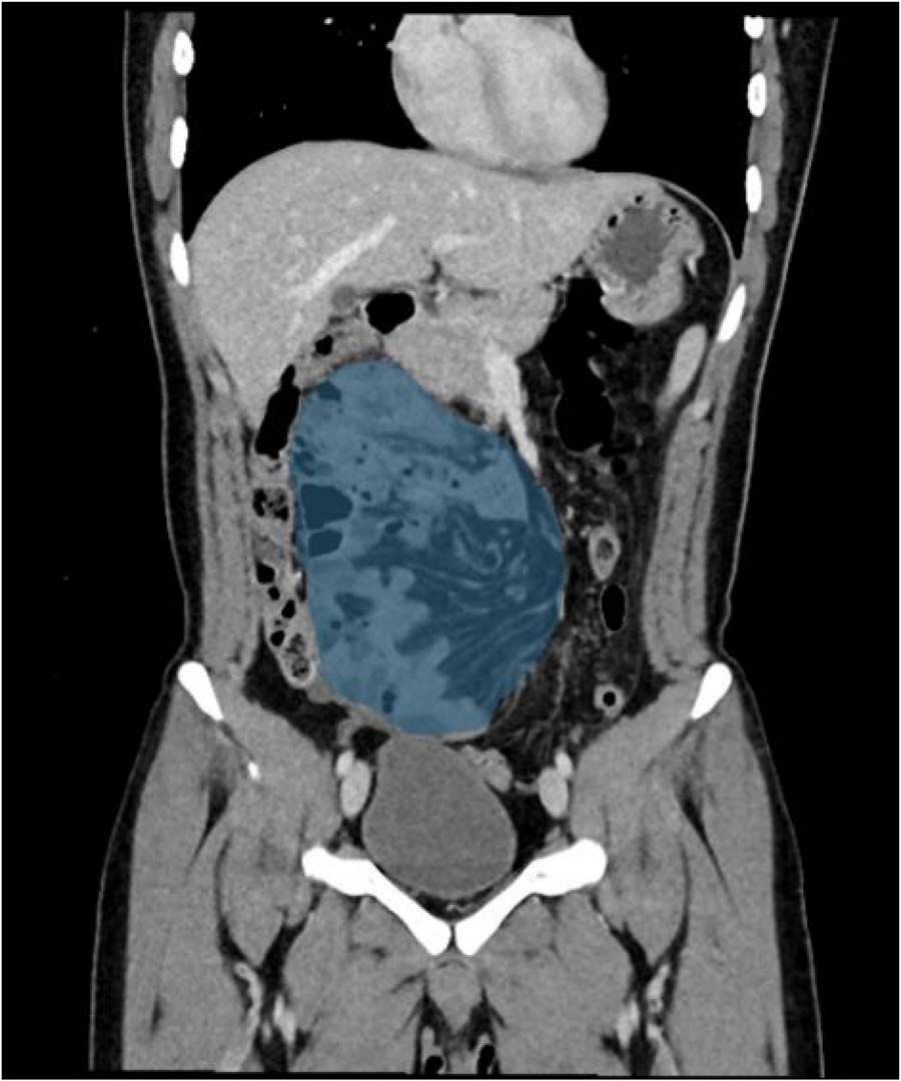
Coronal images showing the clustered small bowel loops in the right hemiabdomen, notice the superior mesentrtic vessels lateral to it the left hemiabdomen.

**Figure 4:**
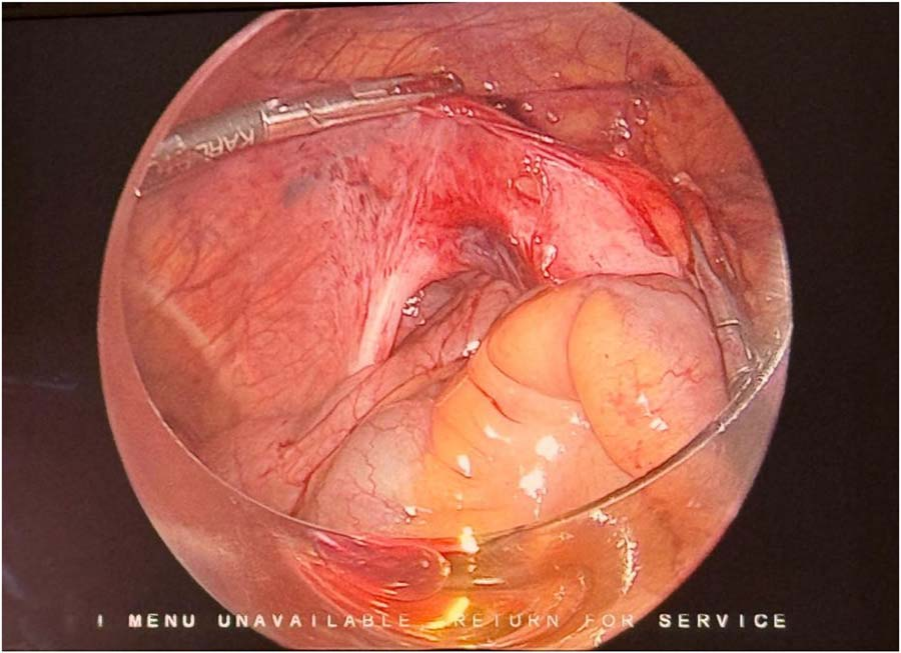
Laparoscopic intraoperative view showing a long, thick vessel-like peritoneal band over the iliac vessels passing cranially toward the mesenteric root, under which small bowel loops have herniated.

A 21-year-old male, medically and surgically free with no known chronic conditions, presented to the Emergency Department with abdominal pain and vomiting. He reported experiencing similar episodes over the past year. Previously, he had received various diagnoses, including gastroenteritis and peptic ulcer disease, and was managed conservatively. On examination, he had mild epigastric tenderness. The patient was hemodynamically stable on presentation, and his complete blood count (CBC) and routine chemistry panel, as shown in Table 1, were unremarkable. Nasogastric decompression was not performed, as the patient did not exhibit significant gastric distension or persistent vomiting requiring decompression. Contrast-enhanced CT of the abdomen demonstrated clustered small bowel loops displacing the superior mesenteric vessels toward the left hemiabdomen (Fig. 1). An additional axial image revealed a thick fibrous band adjacent to the iliac vessels (Fig. 2). Coronal CT images further showed clustered small bowel loops located in the right hemiabdomen, with the superior mesenteric vessels positioned laterally toward the left hemiabdomen (Fig. 3). His symptoms improved upon admission to the surgical ward. Given the congenital nature of the condition, surgical intervention was planned, and a diagnostic laparoscopy was performed. Intraoperative findings were consistent with the CT scan, showing a right iliac fossa defect formed by a long, thick band near the iliac vessels at the root of the mesentery (Fig. 4). The hernia opening was widened, and after medial mobilization of the ascending colon, the small intestine was noted to pass inferior to the third part of the duodenum and lateral to the superior mesenteric vessels, confirming a right paraduodenal hernia. The hernia sac was opened, and the bowel appeared normal with no interloop adhesions. A prophylactic appendectomy was performed due to the unusual position of the appendix after mobilization, to prevent future diagnostic confusion or complications. The entire procedure was completed laparoscopically. Postoperatively, the patient’s symptoms improved, and he was discharged two days after surgery. At the 3-week follow-up, he reported feeling well with only minor postoperative discomfort. He is scheduled for a follow-up visit in 9 months.

**Table 1:** CBC and routine biochemical parameters.

Parameter	Result(s) / Range	Reference range*
CBC		
White blood cell (count)	6.4 × 10^9^/L	4–11 × 10^9^/L
Hemoglobin	15.9 g/dL	13–17 g/dL
Hematocrit	45.7%	40–50%
Platelets	220 × 10^9^/L	150–400 × 10^9^/L
Chemistry		
Aspartate aminotransferase	14.2 U/L	<40 U/L
Alanine aminotransferase	13.8 U/L	<41 U/L
Alkaline phosphatase	60 U/L	40–129 U/L
Direct bilirubin	3.40 mg/dL	<0.5 mg/dL
Total bilirubin	7.00 mg/dL	0.1–1.2 mg/dL
Sodium	140 mmol/L	135–145 mmol/L
Potassium	4.10 mmol/L	3.5–5.1 mmol/L
Chloride	101 mmol/L	98–106 mmol/L
CO₂ (Bicarbonate)	24 mmol/L	22–29 mmol/L
Calcium	2.23 mmol/L	2.15–2.55 mmol/L
Total protein	75.60 g/L	60–80 g/L
Albumin	48.60 g/L	35–50 g/L
Uric acid	239.0–330.0 μmol/L	200–430 μmol/L
Lipase	37.7 U/L	0–160 U/L

*Reference ranges are based on the institutional laboratory standards.

## Discussion

Right paraduodenal hernias are a very rare type of congenital hernia, representing ~25% of all paraduodenal hernias [4]. In paraduodenal hernias, patients often present with a long-standing history of abdominal pain associated with vomiting and abdominal distention. Many patients have experienced similar episodes since childhood without any significance in the labs and physical examination which can make the diagnosis difficult [5, 6]. It is important to conduct further investigations in such cases, especially when patients present multiple times without a definitive diagnosis. CT scans are available, non-invasive, and play an important role in ruling out internal hernias [7]. Right paraduodenal hernia is described as herniation through Waldeyer’s fossa and occurs due to incomplete or absent rotation of the small bowel during fetal development [6, 8]. This abnormal rotation creates a space below the third part of the duodenum, located behind the small bowel mesentery, which extends downward to the right [8]. The superior mesenteric artery and vein can serve as landmarks for right paraduodenal hernia when they run anteromedial to Waldeyer’s fossa [9]. It is important to differentiate right paraduodenal hernia from midgut volvulus, which is considered a surgical emergency requiring immediate intervention. Midgut volvulus is typically identified on CT by the *whirlpool sign*, representing twisting of the mesenteric vessels around the superior mesenteric artery along with abnormalities of the superior mesenteric artery (SMA) axis [10]. Right paraduodenal hernias are classified into three types based on anatomical and embryological features. Type I is characterized by a normally positioned duodenum and ligament of Treitz, with the hernia orifice located posterior to the superior mesenteric vessels near the jejunocecal isthmus, likely due to incomplete fusion of the ascending colon mesentery with the retroperitoneum. Type II involves absence of the ligament of Treitz, resulting in right-sided displacement of the duodenojejunal junction; the hernia sac contains only the efferent loop and is caused by incomplete rotation of the prearterial midgut. Type III resembles Type II but includes Ladd’s bands, abnormal cecal positioning in the right upper quadrant, and jejunoileal adhesions (Pellerin fusion), indicating severe midgut malrotation [11]. It is important to differentiate between those subtypes to reach the optimal surgical approach. The management of paraduodenal hernia depends on the patient’s clinical presentation. Patients presenting with acute small bowel obstruction require emergent surgical intervention. In contrast, when the diagnosis is established incidentally or in patients with intermittent symptoms, elective surgical repair is recommended, as the lifetime risk of developing small bowel obstruction is reported to be ~50%. If left untreated, paraduodenal hernia may progress to bowel obstruction, ischemia, and perforation, which are associated with high mortality [1, 2, 12, 13]. The surgical intervention includes reduction of the herniated bowel, followed by either closure of the hernia defect with sutures or widening of the defect to prevent future incarceration [14, 15]. The defect can be treated either by primary closure or widening the opening to prevent future incarceration. The choice depends on intraoperative findings; closure is preferred when the bowel is easily reducible, while enlargement of the defect is recommended when reduction is difficult due to a tight neck, edema, or adhesions to allow safe reduction and avoid vascular injury [15]. In our case, the patient had a structure mimicking a vessel, which made the decision to dissect it challenging due to its resemblance to true vascular structures, as shown in Fig. 4. The herniated bowel was successfully reduced, and the patient was managed by widening the hernia defect to facilitate reduction and prevent future incarceration. To the best of our knowledge, no previous case reports in the literature have described a similar finding. Our case demonstrates an abnormal and atypical form of right paraduodenal hernia. Recognizing this variation is crucial, as it helps surgeons distinguish between a peritoneal band and a true vascular structure, thereby facilitating safer intraoperative decision-making. This case further emphasizes the need for heightened suspicion and thorough evaluation in patients with recurrent abdominal symptoms, particularly when imaging or intraoperative findings are atypical.

## Conclusion

We presented a rare case of a young patient with a long standing history of intermittent abdominal symptoms without a definitive diagnosis. In this case, there were many difficulties in reaching the diagnosis, especially due to the unremarkable physical examination and laboratory results. However, it is important to conduct further investigations using imaging tools such as CT scans, which can provide critical details to help in diagnosis. It is important to consider internal hernias in the differential diagnosis of young patients with unexplained, recurrent gastrointestinal symptoms. Early diagnosis and management are essential to reduce mortality and improve patient outcomes.
